# The mutation of Trp64Arg in β3-adrenoreceptor-encoding gene is significantly associated with increased hypertension risk and elevated blood pressure: a meta-analysis

**DOI:** 10.18632/oncotarget.16666

**Published:** 2017-03-29

**Authors:** Hualing Yang, Dongmiao Cai, Qingping Zhu, Dongjin Wu, Qingxiang Wang, Zhanxiang Wang

**Affiliations:** ^1^ Department of Anesthesiology, The First Affiliated Hospital of Xiamen University, Xiamen, Fujian, China; ^2^ The First Clinical Medical College, Fujian Medical University, Fuzhou, Fujian, China; ^3^ Department of Otolaryngology-Head and Neck Surgery, The First Affiliated Hospital of Xiamen University, Xiamen, Fujian, China; ^4^ Department of Neurosurgery, The First Affiliated Hospital of Xiamen University, Xiamen, Fujian, China

**Keywords:** hypertension, β3-adrenoreceptor, polymorphism, blood pressure, meta-analysis

## Abstract

This meta-analysis was implemented to test the association of a missense mutation, Trp64Arg, in β3-adrenoreceptor-encoding gene (*ADRB3*) with both hypertension risk and blood pressure (BP) changes. A systematic search of three publicly-available databases was launched to look for articles published as of December 2016. Qualification appraisal and data extraction were independently done by two researchers. Pooled estimates were expressed as odds ratio (OR) or weighted mean difference (WMD), and their 95% confidence intervals (95% CIs). There were separately 21 (3750/4225 patients/controls) and 17 (6100 subjects) individual studies for hypertension risk and BP changes. Integral analyses revealed that Trp64Arg mutation was associated with the significantly increased risk of hypertension, and particularly, the 64Trp/64Arg heterozygote carriers were 1.23-times more likely to develop hypertension compared with the 64Trp/64Trp homozygote carriers (OR = 1.23, 95% CI: 1.02∼1.46, *P* = 0.021). Publication bias was extremely low for all integral comparisons. In stratified analyses, significance was spotted in populations of Chinese descent, in retrospective studies, in hospital-based studies, in age-matched case-control studies, in studies enrolling patients with mean body mass index < 25 kg/m^2^ and in studies with total sample size ≥ 240. Heterogeneity was improved for most stratified comparisons. Further in hypertensive patients, the 64Trp/64Arg heterozygote carriers had significantly higher systolic (WMD = 0.87 mmHg, 95% CI: 0.39∼1.35, *P* < 0.001) and diastolic (WMD = 0.88 mmHg, 95% CI: 0.59∼1.17, *P* < 0.001) BP than 64Trp/64Trp homozygote carriers. Altogether, ADRB3 gene Trp64Arg mutation was significantly associated with an increased predisposition toward hypertension and elevated systolic/diastolic BP in hypertensive patients, suggesting that Trp64Arg is an important hypertension-susceptibility marker.

## INTRODUCTION

Obesity in humans is well acknowledged as the most important, independent risk factor for hypertension [1]. Speaking in general terms, obesity is the amassment of excessive fat in adipose tissue [2]. One of the principal components in adipose tissue is β3-adrenoreceptor, which is abundantly expressed in brown adipose tissue, as well as in subcutaneous and abnormal white adipose tissue [3, 4]. An important biological property of β3-adrenoreceptor is to mediate the catecholamine-induced thermogenesis and lipolysis in adipose tissue [5, 6]. Another important property involves the enhanced function of sympathetic nervous system, which represents a chief regulator of blood pressure via altering sodium homeostasis and vascular resistance [7, 8]. Echoing from this interlocked relation, it is intriguing to speculate that high expression of β3-adrenoreceptor in adipose tissue would lead to elevated blood pressure and ultimately to the great risk of hypertension.

There is evidence that β3-adrenoreceptor expression is partly under genetic control [9]. The extensively-assessed bi-allelic polymorphism in the gene encoding for β3-adrenoreceptor (*ADRB3*, on chromosome 8p11.23) is rs4994, a missense mutation in the 64th position that substitutes a tryptophan with an arginine, termed as Trp64Arg. This substitution can alter the affinity of β3-adrenoreceptor to norepinephrine and its interaction with G-proteins in adipocytes [10]. Since the year 1996, a growing number of epidemiological studies have tested the association of *ADRB3* gene Trp64Arg polymorphism with hypertension risk and the changes of relevant intermediate phenotypes, such as blood pressure, body mass index (BMI) and fasting insulin [4, 11–14]. However, the results of these studies are not satisfactory, as positive association cannot be reproduced coherently. As a meta-analysis is widely recognized as a useful and reliable method to address this issue, we set out to meta-analyze the association of *ADRB3* gene Trp64Arg polymorphism with both hypertension risk and blood pressure changes using existing publications.

## RESULTS

### Qualified studies

**Figure 1:**
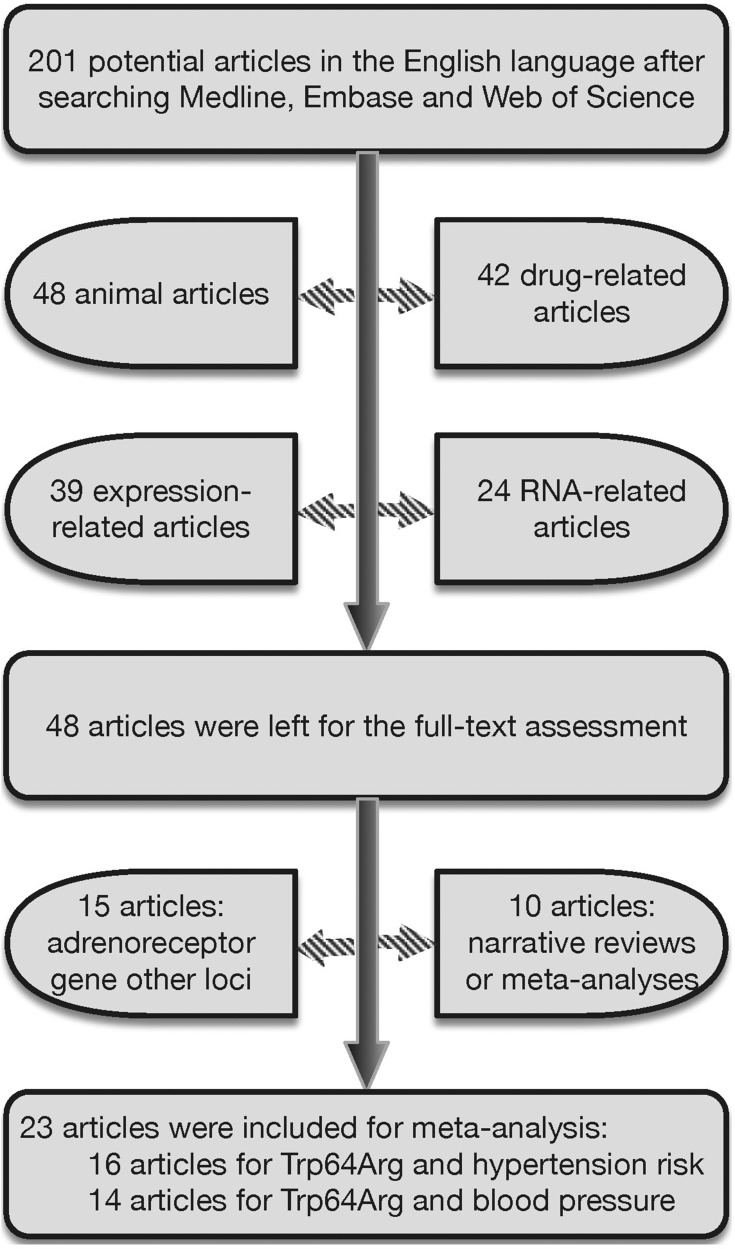
The selection process of qualified publications for this meta-analysis

Using the prescribed keywords, a total of 201 publications in the English language were identified from searching three publicly-available databases. Among them, only 23 publications met preset inclusive criteria [4, 10–31]. The selection process of qualified publications is illustrated in Figure 1. There were 16 publications testing the association of *ADRB3* gene Trp64Arg polymorphism with hypertension risk [10, 11, 14, 16, 17, 19, 20, 23–31], incorporating 21 individual studies (3750 hypertensive patients and 4225 normotensive controls). Fourteen publications were available for the association of Trp64Arg polymorphism with blood pressure changes [4, 10, 12–15, 17, 18, 21–23, 25, 26, 28], incorporating 17 individual studies (6100 subjects). The baseline characteristics of all qualified studies are present in both Supplementary Table 1 and Table 1.

**Table 1:** The baseline characteristics of observational studies for the changes of blood pressure and related intermediate phenotypes

First author		Year	Ethnicity	Design		BP cutoff	Source		Matched		Genotyping	Sample size		Age (years)		Males	
Cases	Controls			Cases	Controls		Cases	Controls			
BMI (kg/m^2^)			SBP (mmHg)		DBP (mmHg)		Glucose (mmol/L)		TG(mmol/L)			TC (mmol/L)		HDLC (mmol/L)		LDLC (mmol/L)	
Cases		Controls	Cases	Controls	Cases	Controls	Cases	Controls	Cases		Controls	Cases	Controls	Cases	Controls	Cases	Controls
Ikegami		1996	Japanese	Retrospective		140/90	Hospital		n.a.		RFLP	126	97	n.a.	n.a.	n.a.	n.a.
Fujisawa		1997	Japanese	Retrospective		160/95	Hospital		NO		RFLP	101	73	57.7	46.2	0.475	0.712
Baba		1998	Japanese	Retrospective		140/90	Hospital		YES		RFLP	37	46	57.0	55.0	0.568	0.565
Tonolo		1999	Caucasian	Retrospective		160/95	Hospital		YES		RFLP	213	281	62.0	61.0	0.460	0.374
Ringel (Men)		2000	Caucasian	Retrospective		n.a.	Hospital		NO		RFLP	86	122	61.6	57.8	1.000	1.000
Ringel (Women)		2000	Caucasian	Retrospective		n.a.	Hospital		NO		RFLP	115	94	64.4	59.8	0.000	0.000
Filigheddu		2004	Caucasian	Retrospective		140/90	Hospital		NO		RFLP	526	192	48.2	66.0	0.580	0.469
Masuo		2005	Japanese	Nested		140/90	Population		n.a.		TaqMan	41	117	n.a.	n.a.	n.a.	n.a.
Nagano		2005	Japanese	Retrospective		140/90	Hospital		n.a.		RFLP	23	66	n.a.	n.a.	0.551	0.551
Kawaguchi		2006	Japanese	Nested		140/90	Population		n.a.		TaqMan	27	28	n.a.	n.a.	n.a.	n.a.
Hui (Men)		2007	Chinese	Retrospective		160/100	Hospital		YES		TaqMan	170	182	51.0	52.0	1.000	1.000
Hui (Women)		2007	Chinese	Retrospective		160/100	Hospital		YES		TaqMan	91	89	51.1	50.4	0.000	0.000
Mo (Obese)		2007	Chinese	Retrospective		140/90	Hospital		YES		RFLP	149	149	57.4	57.8	0.597	0.456
Mo (Non-obese)		2007	Chinese	Retrospective		140/90	Hospital		YES		RFLP	139	149	58.1	57.8	0.576	0.456
Ruixing (Men)		2008	Chinese	Retrospective		140/90	Population		NO		RFLP	287	547	51.2	43.4	1.000	1.000
Ruixing (Women)		2008	Chinese	Retrospective		140/90	Population		NO		RFLP	159	676	55.2	43.8	0.000	0.000
Mirrakhimov		2011	Kyrgyz	Retrospective		n.a.	Population		n.a.		RFLP	87	126	50.7	50.7	0.681	0.681
Chou (Normal)		2012	Chinese	Nested		130/85	Population		n.a.		TaqMan	22	242	n.a.	n.a.	n.a.	n.a.
Chou (Obesity)		2012	Chinese	Nested		130/85	Population		n.a.		TaqMan	110	129	n.a.	n.a.	n.a.	n.a.
Wang		2014	Chinese	Retrospective		140/90	Hospital		YES		TaqMan	1090	700	51.9	51.8	0.606	0.597
Grygiel-Gorniak		2015	Caucasian	Retrospective		140/90	Population		NO		TaqMan	151	120	59.9	58.6	0.000	0.000
n.a.		n.a.	n.a.	n.a.	n.a.	n.a.	n.a.	n.a.	n.a.		n.a.	n.a.	n.a.	n.a.	n.a.	n.a.	n.a.
24.1		22.2	155	122	92	76	n.a.	n.a.	n.a.		n.a.	5.27	5.25	n.a.	n.a.	n.a.	n.a.
23.2		22.6	n.a.	n.a.	n.a.	n.a.	n.a.	n.a.	n.a.		n.a.	n.a.	n.a.	n.a.	n.a.	n.a.	n.a.
30.1		26.7	n.a.	n.a.	n.a.	n.a.	n.a.	n.a.	1.49		1.48	5.90	5.80	1.40	1.50	3.70	3.60
27.4		27.2	n.a.	n.a.	n.a.	n.a.	n.a.	n.a.	n.a.		n.a.	n.a.	n.a.	n.a.	n.a.	n.a.	n.a.
29.0		27.3	n.a.	n.a.	n.a.	n.a.	n.a.	n.a.	n.a.		n.a.	n.a.	n.a.	n.a.	n.a.	n.a.	n.a.
26.9		25.7	157	129	104	80	n.a.	n.a.	n.a.		n.a.	n.a.	n.a.	n.a.	n.a.	n.a.	n.a.
n.a.		n.a.	n.a.	n.a.	n.a.	n.a.	n.a.	n.a.	n.a.		n.a.	n.a.	n.a.	n.a.	n.a.	n.a.	n.a.
n.a.		n.a.	n.a.	n.a.	n.a.	n.a.	n.a.	n.a.	n.a.		n.a.	n.a.	n.a.	n.a.	n.a.	n.a.	n.a.
n.a.		n.a.	n.a.	n.a.	n.a.	n.a.	n.a.	n.a.	n.a.		n.a.	n.a.	n.a.	n.a.	n.a.	n.a.	n.a.
24.4		22.8	171.7	113.2	105.4	70.5	n.a.	n.a.	n.a.		n.a.	5.30	5.09	1.44	1.39	n.a.	n.a.
24.4		22.5	177.1	112.1	70.5	68.2	n.a.	n.a.	n.a.		n.a.	5.70	5.45	1.64	1.58	n.a.	n.a.
27.3		20.2	141.9	108.5	83.4	72.1	5.30	5.28	1.87		1.22	n.a.	n.a.	1.09	1.16	3.19	2.98
21.7		20.2	141.2	108.5	82.4	72.1	5.31	5.28	1.28		1.22	n.a.	n.a.	1.17	1.16	3.01	2.98
22.2		20.9	146.4	119.3	89.8	75.3	n.a.	n.a.	n.a.		n.a.	n.a.	n.a.	n.a.	n.a.	n.a.	n.a.
20.1		21.2	149.4	115.7	84.3	72.1	n.a.	n.a.	n.a.		n.a.	n.a.	n.a.	n.a.	n.a.	n.a.	n.a.
n.a.		n.a.	n.a.	n.a.	n.a.	n.a.	n.a.	n.a.	n.a.		n.a.	n.a.	n.a.	n.a.	n.a.	n.a.	n.a.
n.a.		n.a.	n.a.	n.a.	n.a.	n.a.	n.a.	n.a.	n.a.		n.a.	n.a.	n.a.	n.a.	n.a.	n.a.	n.a.
n.a.		n.a.	n.a.	n.a.	n.a.	n.a.	n.a.	n.a.	n.a.		n.a.	n.a.	n.a.	n.a.	n.a.	n.a.	n.a.
26.5		24.8	141.77	115.66	90.85	75.62	5.43	5.17	2.09		1.43	n.a.	n.a.	1.16	1.40	3.42	3.48
29.9		28.9	157.29	121.02	96.37	77.42	5.46	5.24	1.40		1.23	6.09	5.83	1.66	1.65	3.78	3.61

### Trp64Arg and hypertension risk: integral analyses

In view of a low frequency of 64Arg/64Arg homozygote and to avoid a fluctuated estimate, the risk prediction of Trp64Arg polymorphism for hypertension was investigated separately under the allelic, heterozygote-genotypic and dominant models.

**Figure 2:**
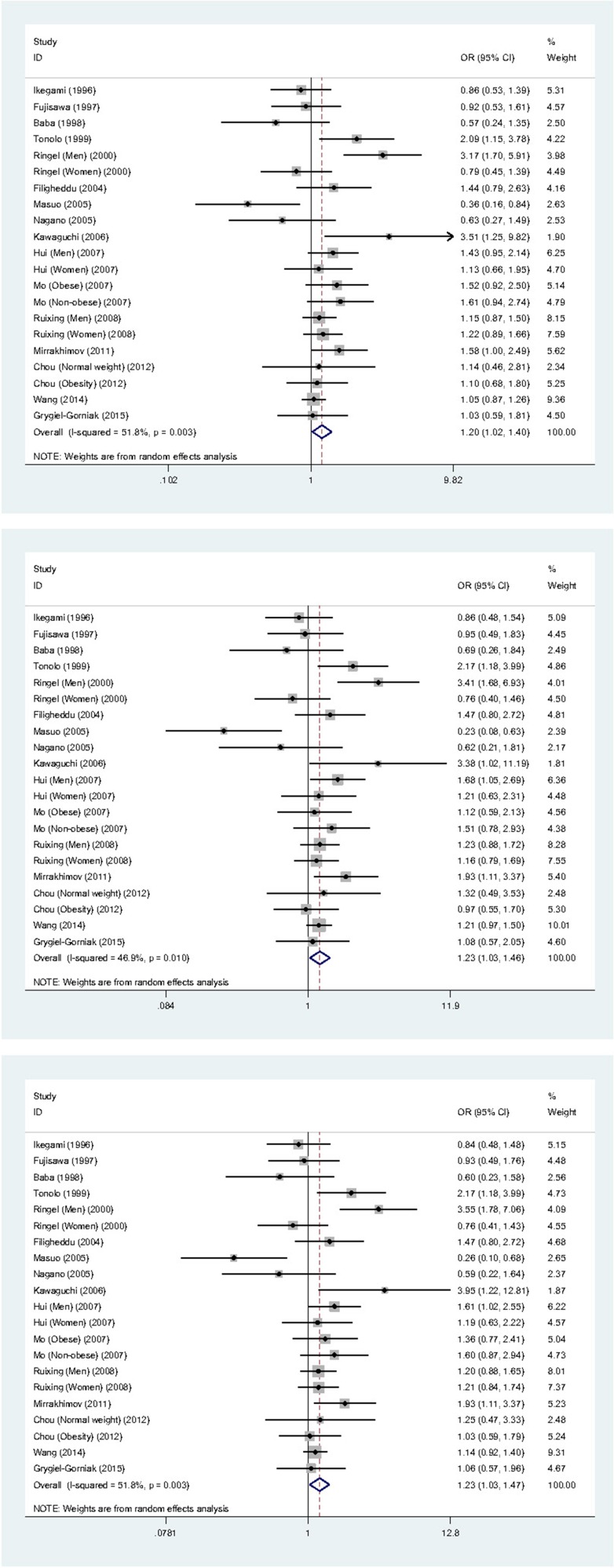
Forest graphs for the integral association of *ADRB3* gene Trp64Arg polymorphism with hypertension risk under the allelic (the upper panel), heterozygote-genotypic (the middle panel) and dominant (the lower panel) models

The mean frequency of 64Arg allele was 15.86% in patients and 14.18% in controls, with the statistical power to detect the difference of being 83.74%. Integral analyses of 21 individual studies revealed that the mutation of *ADRB3* gene Trp64Arg polymorphism was associated with the significantly increased risk of hypertension under three genetic models mentioned above, in company with moderate or marginally significant heterogeneity as gauged by the *I*^2^ statistic (Figure 2). For example, carriers of the 64Trp/64Arg heterozygote were 1.23-times more likely to develop hypertension when compared with those of the 64Trp/64Trp homozygote (OR = 1.23, 95% CI: 1.02 ∼ 1.46, *P* = 0.021), with moderate heterogeneity (*I*^2^ = 46.9%). In addition, the genotype distributions of *ADRB3* gene Trp64Arg polymorphism deviated from the Hardy-Weinberg equilibrium in 4 studies, exclusion of these 4 studies generated the very similar effect estimates, with slightly improved heterogeneity.

**Figure 3:**
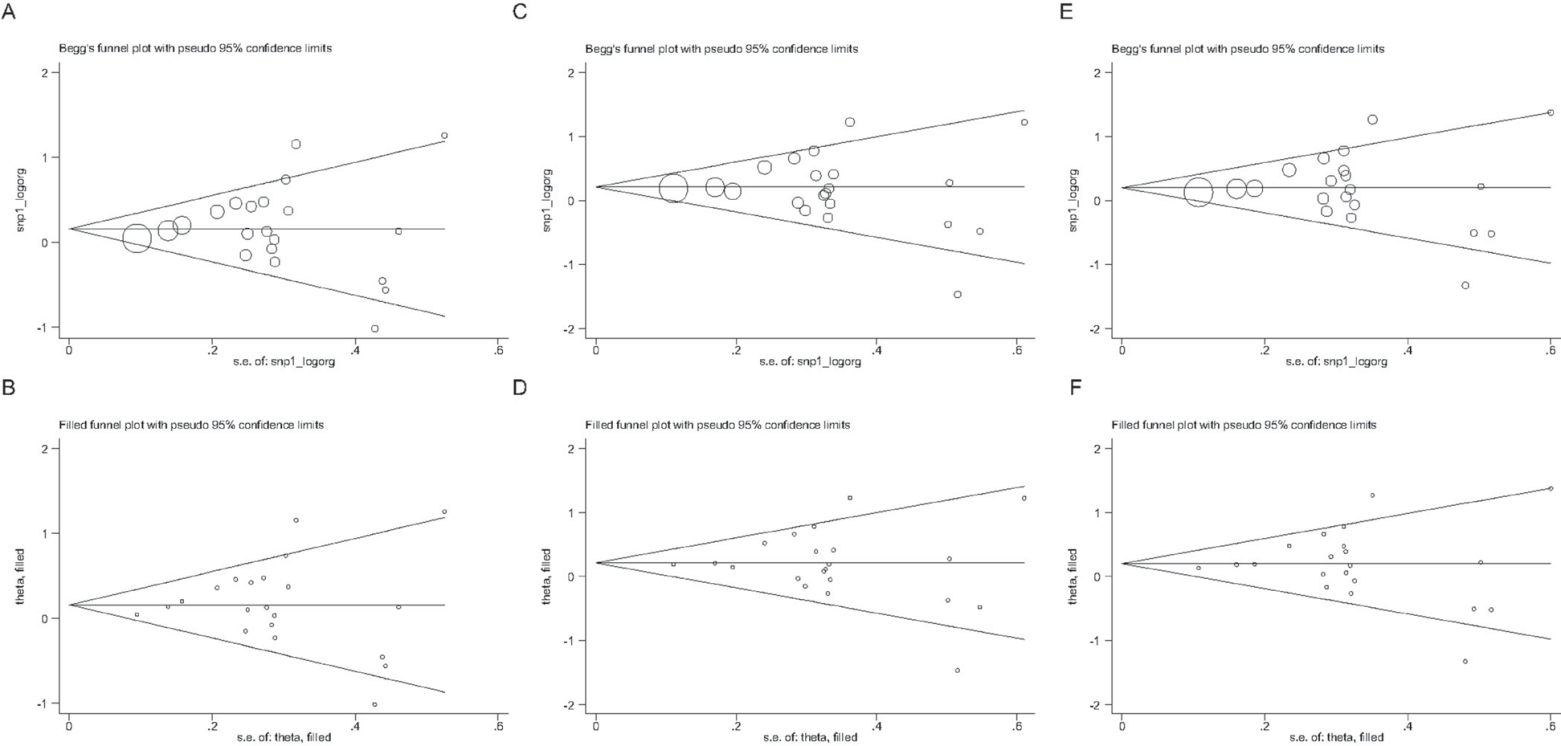
Begg's and filled funnel graphs for the association of ADRB3 gene Trp64Arg polymorphism with hypertension risk under the allelic (**A** and **B** panels), heterozygote-genotypic (**C** and **D** panels) and dominant (**E** and **F** panels) models.

Regarding publication bias, the likelihood of significance was extremely low for the three genetic models mentioned above, as supported by the symmetrical Begg's funnel graphs (Figure 3A, 3C, 3E) and the no-missing-study-reported filled funnel graphs (Figure 3B, 3D, 3F), as well as by the nonsignificant Egger's regression tests for funnel asymmetry (*P* = 0.705, 0.731 and 0.999 for the allelic, heterozygote-genotypic and dominant models, respectively).

### Trp64Arg and hypertension risk: stratified analyses

Because the heterogeneity in integral analyses was moderate or significant, a string of stratified analyses and meta-regression analyses were implemented to look for the possible reasons in explanation of between-study heterogeneity from other demographic and methodological aspects. In stratified analyses, 21 qualified studies were grouped per ethnicity, study design, source of control populations, genotyping methodology, age-matched condition, BMI in patients and total sample size respectively under the allelic, heterozygote-genotypic and dominant models (Table 2).

**Table 2:** The risk prediction of *ADRB3* gene Trp64Arg mutation for hypertension under three genetic models in stratified analyses

Subgroups		Allelic model			Heterozygote-genotypic model			Dominant model		
	Studies (N)	OR, 95% CI	*P*	*I^2^*	OR, 95% CI	*P*	*I^2^*	OR, 95% CI	*P*	*I^2^*
***Ethnicity***										
Caucasian	5	1.49, 0.92 ∼ 2.41	0.103	70.1%	1.54, 0.94 ∼ 2.52	0.085	65.9%	1.54, 0.93 ∼ 2.57	0.095	69.5%
Chinese	9	1.17, 1.04 ∼ 1.31	0.008	0.0%	1.23, 1.07 ∼ 1.41	0.003	0.0%	1.22, 1.07 ∼ 1.39	0.003	0.0%
Japanese	6	0.82, 0.51 ∼ 1.31	0.400	60.3%	0.79, 0.45 ∼ 1.37	0.402	58.7%	0.79, 0.45 ∼ 1.38	0.400	62.4%
***Study design***										
Nested	4	1.08, 0.50 ∼ 2.35	0.842	74.2%	0.97, 0.38 ∼ 2.43	0.943	75.8%	1.03, 0.41 ∼ 2.59	0.948	77.1%
Retrospective	17	1.22, 1.05 ∼ 1.42	0.011	45.7%	1.28, 1.09 ∼ 1.50	0.002	31.6%	1.27, 1.08 ∼ 1.50	0.004	40.9%
*Source of controls*										
Hospital	13	1.22, 0.98 ∼ 1.50	0.072	56.5%	1.27, 1.02 ∼ 1.57	0.030	42.0%	1.26, 1.01 ∼ 1.58	0.043	50.6%
Population	8	1.17, 0.91 ∼ 1.50	0.234	49.8%	1.16, 0.84 ∼ 1.62	0.373	58.3%	1.18, 0.85 ∼ 1.63	0.316	59.2%
***Genotyping methodology***										
RFLP	13	1.25, 1.01 ∼ 1.54	0.037	54.2%	1.27, 1.02 ∼ 1.59	0.034	44.4%	1.28, 1.02 ∼ 1.62	0.032	51.4%
TaqMan	8	1.11, 0.86 ∼ 1.43	0.416	48.6%	1.15, 0.84 ∼ 1.58	0.382	55.8%	1.14, 0.84 ∼ 1.56	0.404	56.1%
***Age-matched condition***										
n.a.	7	1.03, 0.68 ∼ 1.57	0.879	64.1%	1.01, 0.60 ∼ 1.71	0.968	68.0%	1.03, 0.61 ∼ 1.74	0.914	69.5%
NO	7	1.22, 0.94 ∼ 1.58	0.130	54.1%	1.25, 0.95 ∼ 1.65	0.115	46.3%	1.25, 0.94 ∼ 1.67	0.119	52.5%
YES	7	1.28, 1.01 ∼ 1.62	0.038	45.7%	1.32, 1.10 ∼ 1.58	0.003	5.8%	1.33, 1.07 ∼ 1.64	0.011	25.5%
***Mean BMI in patients***										
BMI < 25 kg/m^2^	7	1.19, 1.02 ∼ 1.39	0.029	0.0%	1.24, 1.03 ∼ 1.49	0.025	0.0%	1.23, 1.03 ∼ 1.48	0.022	0.0%
BMI ≥ 25 kg/m^2^	14	1.22, 0.96 ∼ 1.55	0.105	63.6%	1.23, 1.03 ∼ 1.60	0.136	61.3%	1.24, 0.95 ∼ 1.63	0.118	64.4%
***Total sample size***										
< 240	11	1.06, 0.76 ∼ 1.48	0.725	68.5%	1.07, 0.72 ∼ 1.57	0.746	67.4%	1.07, 0.72 ∼ 1.59	0.736	70.5%
≥ 240	10	1.20, 1.07 ∼ 1.35	0.002	0.0%	1.29, 1.13 ∼ 1.48	<0.001	0.0%	1.27, 1.11 ∼ 1.44	<0.001	0.0%

Abbreviations: OR, odds ratio; 95% CI, 95% confidence interval; RFLP, restriction fragment length polymorphism; BMI, body mass index; n.a., not available.

When all studies were grouped by ethnicity, significance was only revealed in populations of Chinese descent (*n* = 9) for the association of Trp64Arg polymorphism with hypertension risk under all three genetic models, and no evidence of heterogeneity occurred (*I*^2^ = 0.0% for all models). By study design, the risk prediction was only significant in retrospective studies (*n* = 17), with moderate heterogeneity. By source of controls, effect-size estimates were potentiated in hospital-based studies (*n* = 13) relative to population-based studies (*n* = 8), especially under the heterozygote-genotypic and dominant models, and there was moderate evidence of heterogeneity. By genotyping methodology, studies using RFLP (restriction fragment length polymorphism) technique reported a significant association of Trp64Arg mutation with hypertension under all three genetic models, and this association was biased by significant heterogeneity. By age-matched condition, the mutation of Trp64Arg polymorphism was significantly associated with the increased risk of hypertension in studies involving age-matched hypertensive patients and normotensive controls (*n* = 7), and the probability of heterogeneity was considerably improved under the heterozygote-genotypic (*I*^2^ = 5.8%) and dominant (*I*^2^ = 25.5%) models. By mean BMI in patients at a cutoff value of 25 kg/m^2^, the risk prediction of Trp64Arg polymorphism for hypertension was significant when analysis was restricted to the studies enrolling patients with mean BMI < 25 kg/m^2^ (*n* = 7) under the allelic (OR=1.19, 95% CI: 1.02 ∼ 1.39, *P* = 0.029), heterozygote-genotypic (OR=1.24, 95% CI: 1.03 ∼ 1.49, *P* = 0.025) and dominant (OR=1.23, 95% CI: 1.03 ∼ 1.48, *P* = 0.022), without heterogeneity (all *I*^2^ = 0.0%). Finally, grouping studies by total sample size at the medium cutoff value of 240 found a robust association in larger studies (*n* = 10) between Trp64Arg polymorphism and hypertension risk under the allelic (OR=1.20, 95% CI: 1.07 ∼ 1.35, *P* = 0.001), heterozygote-genotypic (OR=1.29, 95% CI: 1.13 ∼ 1.48, *P* < 0.001) and dominant (OR=1.27, 95% CI: 1.11 ∼ 1.44, *P* < 0.001) models, respectively, and there was no detectable heterogeneity (all *I*^2^ = 0.0%).

Further meta-regression analyses, when modeling the baseline (age, gender, BMI, smoking) and clinical (FBG, triglycerides, total cholesterol, HDLC, LDLC) characteristics, failed to detect any significant contributions on the risk prediction of Trp64Arg polymorphism for hypertension under all three genetic models (all *P* > 0.05) (data not shown).

### Trp64Arg and intermediate phenotype changes

Because of the close relation of β3-adrenoreceptor with obesity and lipolysis, besides blood pressure, the levels of other related intermediate phenotypes including BMI, FBG, fasting insulin, total cholesterol and HDLC were also compared across Trp64Arg genotypes. Considering a low frequency of the 64Arg/64Arg homozygote, the 64Trp/64Arg heterozygote is often combined with the 64Arg/64Arg homozygote when assessing the changes of these phenotypes across these genotypes. To avoid deviations from inadequate sample sizes, blood pressure and related intermediate phenotypes were only compared between carriers of the 64Trp/64Trp and 64Trp/64Arg genotypes (Table 3). Integral analyses of 17 individual studies failed to detect any significant changes in blood pressure and related intermediate phenotypes between the 64Trp/64Trp and 64Trp/64Arg genotypes. Further grouping 17 studies by hypertension status found that in hypertensive patients, the 64Trp/64Arg heterozygote carriers had significantly higher SBP (WMD=0.87 mmHg, 95% CI: 0.39 ∼ 1.35, *P* < 0.001) and DBP (WMD=0.88 mmHg, 95% CI: 0.59 ∼ 1.17, *P <* 0.001) levels than those with the 64Trp/64Trp homozygote, with no detectable heterogeneity (both *I*^2^ = 0.0%). By contrast, BMI was 3.00 kg/m^2^ higher in normotensive controls possessing the 64Trp/64Arg heterozygote than the 64Trp/64Trp homozygote (WMD=3.00, 95% CI: 0.35 ∼ 5.66, *P* = 0.026), without heterogeneity (*I*^2^ = 0.0%), while it was 0.70 kg/m^2^ lower in hypertensive patients. As for total cholesterol, normotensive controls with the 64Trp/64Arg heterozygote had a lower level than those with the 64Trp/64Trp homozygote (WMD=−0.39 mmol/L, 95% CI: −0.73 ∼ −0.05, *P* = 0.026), without heterogeneity (*I*^2^ = 0.0%). No significant changes were observed in FBG, fasting insulin and HDLC levels across Trp64Arg genotypes (all *P* > 0.05).

**Table 3:** Pooled changes of blood pressure and related intermediate phenotypes between carriers of the 64Trp/64Trp and 64Trp/64Arg genotypes

Phenotypes	Groups	Studies (N)	WMD	95% CI	*P*	*I^2^*
SBP (mmHg)	All studies	16	1.67	−0.48 ∼ 3.82	0.128	80.7%
	Studies in hypertensives	6	0.87	0.39 ∼ 1.35	< 0.001	0.0%
DBP (mmHg)	All studies	16	0.29	−0.28 ∼ 0.87	0.317	15.3%
	Studies in hypertensives	6	0.88	0.59 ∼ 1.17	< 0.001	0.0%
BMI (kg/m^2^)	All studies	14	−0.06	−0.59 ∼ 0.48	0.838	83.4%
	Studies in hypertensives	5	−0.70	−1.24 ∼ -0.17	0.010	42.1%
FBG (mmol/L)	All studies	6	0.01	−0.06 ∼ 0.07	0.835	0.0%
	Studies in hypertensives	3	0.03	−0.08 ∼ 0.15	0.574	12.0%
Fasting insulin (pmol/L)	All studies	11	−0.94	−7.00 ∼ 5.12	0.762	71.1%
	Studies in hypertensives	3	−2.07	−11.54 ∼ 7.39	0.668	0.0%
Total cholesterol (mmol/L)	All studies	12	−0.03	−0.12 ∼ 0.05	0.433	49.9%
	Studies in hypertensives	4	0.00	−0.04 ∼ 0.04	0.891	2.8%
HDLC (mmol/L)	All studies	9	−0.01	−0.06 ∼ 0.03	0.580	64.9%
	Studies in hypertensives	4	−0.01	−0.08 ∼ 0.07	0.879	50.7%

Abbreviations: WMD, weighted mean difference; 95% CI, 95% confidence interval; SBP, systolic blood pressure; DBP, diastolic blood pressure; BMI, body mass index; FBG, fasting blood glucose; HDLC, high-density lipoprotein cholesterol.

## DISCUSSION

To the best of our knowledge, this is to date the first meta-analysis to test the association of *ADRB3* gene Trp64Arg polymorphism with both hypertension risk and blood pressure changes. Most notably, the mutation of Trp64Arg polymorphism was significantly associated not only with an increased predisposition toward hypertension, but also with elevated SBP and DBP in hypertensive patients, suggesting that Trp64Arg polymorphism might represent an important genetic marker in the development of hypertension.

Human β3-adrenoreceptor is a member of the family of 7-transmembrance G-protein-coupled receptors. It is widely recognized that the β3-adrenoreceptor is indirectly involved in the etiology of hypertension through reducing lipolysis and activating the sympathetic nervous system [25, 32]. Numerous studies have suggested that hypertension is partly under genetic control, and therefore to look for candidate genetic markers responsible for hypertension predisposition and/or blood pressure changes is justifiable [33, 34]. Given the biological importance of β3-adrenoreceptor, it would be tempting to speculate that certain genetic alterations of its coding gene, *ADRB3*, might play a critical role in the regulation of blood pressure and ultimately predispose to the occurrence of hypertension.

The genomic sequence of *ADRB3* gene is covered with many polymorphic loci, and a missense mutation, Trp64Arg, in its first exon has been widely evaluated in susceptibility to hypertension and other complications in populations from different regions of the world [20, 24, 35]. Some studies have reported the increased risk of hypertension associated with the 64Arg allele [10, 17, 26], whereas others have found an apparently conflicting association [25, 31]. This controversy could be attributed to the differences in genetic background, study design, source of study subjects, sample size and so on [36]. So, attempts to account for these differences have justified the necessity of undertaking a systematic meta-analysis, just as the present study did.

Our integral analyses demonstrated that the mutation of Trp64Arg polymorphism was associated with the significantly increased risk of hypertension, and this association was further substantiated in age-matched case-control studies and in larger studies, irrespective of three genetic models under investigation, highlighting the robustness of our meta-analytical findings. However, in many cases, complex diseases like hypertension are complicated by genetic heterogeneity [37]. As demonstrated by our ethnicity-stratified analyses, there was no exception for *ADRB3* gene Trp64Arg polymorphism either. The risk prediction of this polymorphism for hypertension was spotted in populations of Chinese descent, whereas the Trp64Arg mutation seemed to act as a protective factor against the occurrence of hypertension in Japanese, albeit nonsignificantly. The possible reasons for such divergence could be due to differences in statistical power, genetic background, environmental exposure or dietary habit. Hence, the presence of genetic heterogeneity might undermine the replication of association between *ADRB3* gene and hypertension. In addition, our findings also suggested that the differences in study design, genotyping methodology, age-matched condition, obesity and total sample size might be the potential sources of between-study heterogeneity. Altogether, our findings not only confirmed the susceptible role of *ADRB3* gene Trp64Arg polymorphism in the development of hypertension, but also aid in explaining the conflicting patterns of the association seen in prior studies.

Another important finding of this meta-analysis was the significant changes in blood pressure across *ADRB3* gene Trp64Arg genotypes. In fact, the heterozygous carriers of Trp64Arg polymorphism were found to have significantly higher SBP and DBP than those with the 64Trp/64Trp genotype in hypertensive patients, which can serve as an apt explanation for the risk-conferring association of the 64Arg allele with hypertension, as discussed above. It is generally believed that the sympathetic nervous system represents a major regulator of blood pressure through alterations in sodium homeostasis and vascular resistance [38, 39]. So, we speculate that the mutation of Trp64Arg polymorphism might impact on the function of β3-adrenoreceptor, which can further activate sympathetic nervous system, regulate blood pressure and ultimately precipitate the development of hypertension.

### Study limitations

This meta-analysis is based on observational data from genetic association studies, and the potential causality cannot be handled. Moreover, only the English publications were meta-analyzed, and doing so might raise a possible selection bias. However, as manifested by the Begg's funnel graph, the filled funnel graph and the Egger's regression asymmetry test, publication bias was unlikely to be a perplexing issue. In addition, only one missense mutation was analyzed in the present study, and it is highly encouraged to incorporate more polymorphisms in close linkage disequilibrium with Trp64Arg polymorphism in *ADRB3* gene and other adrenoreceptor genes, such as *ADRB1* and *ADRB2* genes, pending sufficient available publications to investigate their joint impact on blood pressure and hypertension risk. Also, because β3-adrenoreceptor is a principal component of adipose tissue, we only stratified the studies according to mean BMI, an indication of general obesity, and did not assess another important index of abdominal obesity, waist-to-hip ratio due to insufficient eligible studies. Furthermore, this meta-analysis was limited by inadequate sample size, especially in some subgroups. We concede that replication of our findings in other well-designed studies with a larger sample size will add additional evidence to our existing knowledge on a contributing role of *ADRB3* gene to the etiology of hypertension.

## MATERIALS AND METHODS

### Search information

To be as comprehensive as possible, a systematic search of three publicly-available electronic databases, Medline, Embase (Excerpta Medica Database) and Web of Science, was launched to look for articles published as of December 14, 2016. The prescribed MeSH key terms incorporated “β3-adrenoreceptor” or “β3-adrenergic receptor” or “beta3-adrenoreceptor” or “beta3-adrenergic receptor” or “β3-adrenoreceptor” or “ADRB3” or “3-BAR” or “beta3-AR” in the Title/Abstract, annexed with “hypertension” or “hypertensive” or “blood pressure” in the Title and “genetic” or “polymorphism” or “SNP” or “variant” or “mutation” or “genotype” or “allele” in the Title/Abstract. Search results were primarily confined to the English-only articles involving human beings, irrespective of races or ethnicities or study designs. Search scope was further expanded to include reference lists of the keywords-indexed results to avoid possible omissions. Search process was done by two researchers - Hualing Yang and Dongmiao Cai, and the final publication list was based on congregated results of two researchers by removing duplications with the EndNote citation manager software version X8 (Thomson Reuters).

### Inclusive criteria

Inclusive criteria were determined by all listed authors of this study, from the following three respects: (i) study design: either retrospective or nested; (ii) genetic data: the genotype or allele distributions of *ADRB3* gene Trp64Arg polymorphism between hypertensive patients and normotensive controls or the mean and standard deviation of systolic blood pressure (SBP) and diastolic blood pressure (DBP) in mmHg, and when available other related intermediate phenotypes, including BMI, fasting blood glucose (FBG), fasting insulin, total cholesterol and HDLC across Trp64Arg genotypes; (iii) the genotypes of Trp64Arg polymorphism distinguished by validated methods. Two researchers - Hualing Yang and Dongmiao Cai, independently filtrated eligible articles from all keywords- and references-indexed search results per the inclusive criteria formulated above, and they also fixed all discrepancies with consensus.

### Data extraction

A data-extraction table in Excel version 2016 was delineated by all listed authors, and extraction process was implemented by the same two researchers - Hualing Yang and Dongmiao Cai. The extracted data of interest contained the first author's family name, year of publication, country of samples collected, race or ethnicity of study subjects, study design, source of control populations, age-matched condition between patients and controls, genotyping methodology, sample size, age, gender, BMI, smoking, SBP, DBP, FBG, triglycerides, total cholesterol, high-density lipoprotein cholesterol (HDLC), low-density lipoprotein cholesterol, the genotype or allele distributions of Trp64Arg polymorphism between patients and controls, and the mean and standard deviation of SBP, DBP, BMI, FBG, fasting insulin, total cholesterol and HDLC levels across Trp64Arg genotypes, where existing. Two independently-extracted tables were compared for divergence, and any disagreement was resolved through discussion.

### Statistical analyses

The risk prediction of *ADRB3* gene Trp64Arg polymorphism for hypertension was signified as odds ratio (OR) and its 95% confidence interval (95% CI), and the changes of blood pressure and other related intermediate phenotypes were signified as weighted mean difference (WMD) and its 95% CI. Individual effect-size estimates were combined under random-effects model adopting the method developed by DerSimonian R and Laird N [40]. Statistical heterogeneity of individual studies was expressed as the *I*^2^ statistic, a per cent number that can be interpreted as low (*I*^2^ ≤ 25%), moderate (*I*^2^ > 25% and *I*^2^ < 50%) or high (*I*^2^ ≥ 50%) [41]. Other sources of heterogeneity from demographic and methodological aspects were probed by stratified analyses and meta-regression analyses. Publication bias was gauged by the Begg's funnel graph, the filled funnel graph and the Egger's regression asymmetry test [42]. The probability for the Egger's test was significant at 10% or less. In the presence of missing studies, an unbiased estimate was derived by the trim-and-fill method. Data were statistically analyzed by the Meta-analysis module [43] in the Stata/SE software version 14.1 for the Windows. Study power was estimated by the “sampsi” order in the Stata/SE software.

## CONCLUSIONS

The collective findings of this meta-analysis demonstrated that the mutation of Trp64Arg polymorphism was significantly associated not only with an increased predisposition toward hypertension, but also with elevated SBP and DBP in hypertensive patients, suggesting that Trp64Arg polymorphism might represent an important genetic marker in the development of hypertension. These findings will potentially further our understanding for the contributing role of *ADRB3* gene Trp64Arg polymorphism in blood pressure regulation and in the pathogenesis of hypertension.

## SUPPLEMENTARY MATERIALS FIGURES AND TABLES





## References

[1] Hall JE, do Carmo JM, da Silva AA, Wang Z, Hall ME (2015). Obesity-induced hypertension: interaction of neurohumoral and renal mechanisms. Circ Res.

[2] Cabia B, Andrade S, Carreira MC, Casanueva FF, Crujeiras AB (2016). A role for novel adipose tissue-secreted factors in obesity-related carcinogenesis. Obes Rev.

[3] Deiuliis JA, Liu LF, Belury MA, Rim JS, Shin S, Lee K (2010). Beta(3)-adrenergic signaling acutely down regulates adipose triglyceride lipase in brown adipocytes. Lipids.

[4] Erhardt E, Czako M, Csernus K, Molnar D, Kosztolanyi G (2005). The frequency of Trp64Arg polymorphism of the beta3-adrenergic receptor gene in healthy and obese Hungarian children and its association with cardiovascular risk factors. Eur J Clin Nutr.

[5] Philipson LH (2002). beta-Agonists and metabolism. J Allergy Clin Immunol.

[6] Mund RA, Frishman WH (2013). Brown adipose tissue thermogenesis: beta3-adrenoreceptors as a potential target for the treatment of obesity in humans. Cardiol Rev.

[7] Dessy C, Balligand JL (2010). Beta3-adrenergic receptors in cardiac and vascular tissues emerging concepts and therapeutic perspectives. Adv Pharmacol.

[8] Gasparetti AL, Alvarez-Rojas F, de Araujo EP, Hirata AE, Saad MJ, Velloso LA (2005). beta3-Adrenergic-dependent and -independent mechanisms participate in cold-induced modulation of insulin signal transduction in brown adipose tissue of rats. Pflugers Arch.

[9] Campagna F, Montali A, Baroni MG, Maria AT, Ricci G, Antonini R, Verna R, Arca M (2002). Common variants in the lipoprotein lipase gene, but not those in the insulin receptor substrate-1, the beta3-adrenergic receptor, and the intestinal fatty acid binding protein-2 genes, influence the lipid phenotypic expression in familial combined hyperlipidemia. Metabolism.

[10] Mirrakhimov AE, Kerimkulova AS, Lunegova OS, Moldokeeva CB, Zalesskaya YV, Abilova SS, Sovhozova NA, Aldashev AA, Mirrakhimov EM (2011). An association between TRP64ARG polymorphism of the B3 adrenoreceptor gene and some metabolic disturbances. Cardiovasc Diabetol.

[11] Ikegami H, Yamato E, Fujisawa T, Hamada Y, Fujioka Y, Rakugi H, Higaki J, Murakami H, Shimamoto K, Ogihara T (1996). Analysis of candidate genes for insulin resistance in essential hypertension. Hypertens Res.

[12] Buettner R, Schaffler A, Arndt H, Rogler G, Nusser J, Zietz B, Enger I, Hugl S, Cuk A, Scholmerich J, Palitzsch KD (1998). The Trp64Arg polymorphism of the beta 3-adrenergic receptor gene is not associated with obesity or type 2 diabetes mellitus in a large population-based Caucasian cohort. J Clin Endocrinol Metab.

[13] Iwamoto Y, Ohishi M, Yuan M, Tatara Y, Kato N, Takeya Y, Onishi M, Maekawa Y, Kamide K, Rakugi H (2011). beta-Adrenergic receptor gene polymorphism is a genetic risk factor for cardiovascular disease: a cohort study with hypertensive patients. Hypertens Res.

[14] Grygiel-Gorniak B, Kaczmarek E, Mosor M, Przyslawski J, Nowak J (2015). Association of PPAR-gamma2 and beta3-AR Polymorphisms With Postmenopausal Hypertension. J Clin Hypertens (Greenwich).

[15] Yuan M, Ohishi M, Ito N, Sugimoto K, Takagi T, Terai M, Katsuya T, Rakugi H, Wu Z, Ogihara T (2006). Genetic influences of beta-adrenoceptor polymorphisms on arterial functional changes and cardiac remodeling in hypertensive patients. Hypertens Res.

[16] Wang L, Zhang B, Li M, Li C, Liu J, Liu Y, Wang Z, Zhou J, Wen S (2014). Association between single-nucleotide polymorphisms in six hypertensive candidate genes and hypertension among northern Han Chinese individuals. Hypertens Res.

[17] Tonolo G, Melis MG, Secchi G, Atzeni MM, Angius MF, Carboni A, Ciccarese M, Malavasi A, Maioli M (1999). Association of Trp64Arg beta 3-adrenergic-receptor gene polymorphism with essential hypertension in the Sardinian population. J Hypertens.

[18] Strazzullo P, Iacone R, Siani A, Cappuccio FP, Russo O, Barba G, Barbato A, D'Elia L, Trevisan M, Farinaro E (2001). Relationship of the Trp64Arg polymorphism of the beta3-adrenoceptor gene to central adiposity and high blood pressure: interaction with age. Cross-sectional and longitudinal findings of the Olivetti Prospective Heart Study. J Hypertens.

[19] Ruixing Y, Jinzhen W, Shangling P, Weixiong L, Dezhai Y, Yuming C (2008). Sex differences in environmental and genetic factors for hypertension. Am J Med.

[20] Ringel J, Kreutz R, Distler A, Sharma AM (2000). The Trp64Arg polymorphism of the beta3-adrenergic receptor gene is associated with hypertension in men with type 2 diabetes mellitus. Am J Hypertens.

[21] Pamies-Andreu E, Garcia-Lozano R, Palmero-Palmero C, Garcia-Morillo S, Alonso-Arcas A, Stiefel P, Carneado de la Fuente J, Villar J (2000). Genetic variation in the beta-3-adrenergic receptor in essential hypertension. Life Sci.

[22] Oizumi T, Daimon M, Saitoh T, Kameda W, Yamaguchi H, Ohnuma H, Igarashi M, Eguchi H, Manaka H, Tominaga M, Kato T (2001). Genotype Arg/Arg, but not Trp/Arg, of the Trp64Arg polymorphism of the beta(3)-adrenergic receptor is associated with type 2 diabetes and obesity in a large Japanese sample. Diabetes Care.

[23] Nagano T, Matsuda Y, Tanioka T, Yoshioka T, Hiroi T, Yoshikawa K, Okabe K, Osaka K, Nagamine I, Takasaka Y (2005). No association of the Trp 64 Arg mutation of the beta3-adrenergic receptor gene with obesity, type 2 diabetes mellitus, hyperlipidemia, and hypertension in Japanese patients with schizophrenia. J Med Invest.

[24] Mo W, Zhang GG, Yang TL, Dai XP, Li HH, Zeng H, Liu J, Tan YM, Zhou HH, Liu ZQ (2007). The genetic polymorphisms of beta3-adrenergic receptor (AR) Trp64Arg and beta2-AR Gln27Glu are associated with obesity in Chinese male hypertensive patients. Clin Chem Lab Med.

[25] Masuo K, Katsuya T, Fu Y, Rakugi H, Ogihara T, Tuck ML (2005). Beta2- and beta3-adrenergic receptor polymorphisms are related to the onset of weight gain and blood pressure elevation over 5 years. Circulation.

[26] Kawaguchi H, Masuo K, Katsuya T, Sugimoto K, Rakugi H, Ogihara T, Tuck ML (2006). beta2- and beta3-Adrenoceptor polymorphisms relate to subsequent weight gain and blood pressure elevation in obese normotensive individuals. Hypertens Res.

[27] Hui P, Nakayama T, Morita A, Sato N, Hishiki M, Saito K, Yoshikawa Y, Tamura M, Sato I, Takahashi T, Soma M, Izumi Y, Ozawa Y (2007). Common single nucleotide polymorphisms in Japanese patients with essential hypertension: aldehyde dehydrogenase 2 gene as a risk factor independent of alcohol consumption. Hypertens Res.

[28] Fujisawa T, Ikegami H, Yamato E, Hamada Y, Kamide K, Rakugi H, Higaki J, Murakami H, Shimamoto K, Ogihara T (1997). Trp64Arg mutation of beta3-adrenergic receptor in essential hypertension: insulin resistance and the adrenergic system. Am J Hypertens.

[29] Filigheddu F, Reid JE, Troffa C, PinnaParpaglia P, Argiolas G, Testa A, Skolnick M, Glorioso N (2004). Genetic polymorphisms of the beta-adrenergic system: association with essential hypertension and response to beta-blockade. Pharmacogenomics J.

[30] Chou YC, Tsai CN, Lee YS, Pei JS (2012). Association of adrenergic receptor gene polymorphisms with adolescent obesity in Taiwan. Pediatr Int.

[31] Baba T, Nakajima S, Yajima Y (1998). Beta3-adrenergic receptor gene polymorphism is not associated with hypertension in NIDDM patients without nephropathy. Horm Metab Res.

[32] Sheng LJ, Ruan CC, Ma Y, Chen DR, Kong LR, Zhu DL, Gao PJ (2016). Beta3 adrenergic receptor is involved in vascular injury in deoxycorticosterone acetate-salt hypertensive mice. FEBS Lett.

[33] Niu W, Wu S, Zhang Y, Li W, Ji K, Gao P, Zhu D (2010). Validation of genetic association in apelin-AGTRL1 system with hypertension in a larger Han Chinese population. J Hypertens.

[34] Austin ED, Loyd JE (2014). The genetics of pulmonary arterial hypertension. Circ Res.

[35] Zafarmand MH, van der Schouw YT, Grobbee DE, de Leeuw PW, Bots ML (2008). T64A polymorphism in beta3-adrenergic receptor gene (ADRB3) and coronary heart disease: a case-cohort study and meta-analysis. J Intern Med.

[36] Kong H, Li X, Zhang S, Guo S, Niu W (2013). The beta1-adrenoreceptor gene Arg389Gly and Ser49Gly polymorphisms and hypertension: a meta-analysis. Mol Biol Rep.

[37] Kato N (2012). Ethnic differences in genetic predisposition to hypertension. Hypertens Res.

[38] Padmanabhan S, Caulfield M, Dominiczak AF (2015). Genetic and molecular aspects of hypertension. Circ Res.

[39] Kelly TN, He J (2012). Genomic epidemiology of blood pressure salt sensitivity. J Hypertens.

[40] DerSimonian R, Laird N (1986). Meta-analysis in clinical trials. Control Clin Trials.

[41] Higgins JP, Thompson SG, Deeks JJ, Altman DG (2003). Measuring inconsistency in meta-analyses. BMJ.

[42] Mavridis D, Salanti G (2014). How to assess publication bias: funnel plot, trim-and-fill method and selection models. Evid Based Ment Health.

[43] Palmer T, Sterne J (2016). Meta-Analysis in Stata: An Updated Collection from the Stata Journal.

