# Supplementary material for: The mutation of Trp64Arg in β3-adrenoreceptor-encoding gene is significantly associated with increased hypertension risk and elevated blood pressure: a meta-analysis
Source: Oncotarget. 2017 Mar 29;8(28):46480–90. doi: 10.18632/oncotarget.16666 (PMC5542283; doi:10.18632/oncotarget.16666)
Supplement: Supplementary file 1 [file oncotarget-08-46480-s001.pdf]

The mutation of Trp64Arg in β3-adrenoreceptor-encoding gene is significantly associated with increased hypertension risk and elevated blood pressure: a meta-analysis

Supplementary Materials

Supplementary Table 1: The baseline characteristics of all association studies for hypertension risk. See Supplementary_ Table_1
